# Depression with anti-myelin antibodies in the cerebrospinal fluid

**DOI:** 10.1038/s41380-024-02436-5

**Published:** 2024-02-07

**Authors:** Dominique Endres, Lea Berninger, Cornelia Glaser, Luciana Hannibal, Benjamin Berger, Kathrin Nickel, Kimon Runge, Marco Reisert, Horst Urbach, Katharina Domschke, Nils Venhoff, Harald Prüss, Ludger Tebartz van Elst

**Affiliations:** 1 Department of Psychiatry and Psychotherapy, Medical Center - University of Freiburg, Faculty of Medicine, University of Freiburg, Freiburg, Germany; 2 Department of Rheumatology and Clinical Immunology, Medical Center - University of Freiburg, Faculty of Medicine, University of Freiburg, Freiburg, Germany; 3 Laboratory of Clinical Biochemistry and Metabolism, Department of General Pediatrics, Adolescent Medicine and Neonatology, Medical Center - University of Freiburg, Faculty of Medicine, University of Freiburg, Freiburg, Germany; 4 Department of Neurology, Medical Center - University of Freiburg, Faculty of Medicine, University of Freiburg, Freiburg, Germany; 5 Department for Neurology, Helios Clinic Pforzheim, Pforzheim, Germany; 6 Department of Diagnostic and Interventional Radiology, Faculty of Medicine, Medical Physics, Medical Center - University of Freiburg, University of Freiburg, Freiburg, Germany; 7 Department of Stereotactic and Functional Neurosurgery, Faculty of Medicine, Medical Center - University of Freiburg, University of Freiburg, Freiburg, Germany; 8 Department of Neuroradiology, Medical Center - University of Freiburg, Faculty of Medicine, University of Freiburg, Freiburg, Germany; 9 Department of Neurology and Experimental Neurology, Charité - Universitätsmedizin Berlin, Berlin, Germany; 10 German Center for Neurodegenerative Diseases (DZNE) Berlin, Berlin, Germany

**Keywords:** Diagnostic markers, Biochemistry

## To the Editor:

According to the biopsychosocial model, major depression usually has multimodal causes [1, 2]. The biological factors associated with the development of depression include stress, (epi-)genetics, monoamines, excitatory/inhibitory neurotransmission, inflammation, myelination, the gut-brain axis, neurotrophins/neurogenesis, mitochondrial processes, and the opioid system [1]. Autoimmune depression [3] as an oligosymptomatic manifestation of autoimmune encephalitis [4] may be associated with anti-central nervous system (CNS) antibodies in the cerebrospinal fluid (CSF) [5]. This article reports a complex case study of a patient with depression, anti-CNS antibodies, and further biological alterations.

A 55-year-old female patient presented with severe depressive syndrome for approximately three years, with core symptoms of depressed mood, reduced energy levels, loss of interest, and severe cognitive deficits (Beck-Depression-Inventory [BDI] score: 42 points). A neurological examination detected no focal neurological deficits. The patient reported a history of repeated alcohol abuse for approximately 20 years but was currently abstinent for several years. Four years earlier, she had developed multisegmental herpes zoster (treated with acyclovir) with persistent neuralgia in the left arm, and she had experienced a traumatic subarachnoid hemorrhage after a fall three years prior to presentation. In addition, she had suffered from restless legs syndrome, which was treated with dopaminergic medication. The patient’s mother had developed dementia in old age, and her father had symptoms of depression.

For approximately three years, the patient had also been experiencing physical symptoms, including joint pain, dyspnea, cough with sputum production, diffuse digestive problems, and skin changes with Raynaud’s syndrome with tricolor phenomenon. These physical complaints were initially assessed as “somatoform” by physicians in charge.

A recent systematic diagnostic work-up revealed a suspected mild polymyositis-scleroderma overlap syndrome with anti-PM-Scl (+++), anti-PM-Scl 100 (++), and anti-Mi2 beta (+) antibodies along with Raynaud’s syndrome (with a small reduction in perfusion in the fingers and toes and megacapillaries) as well as nonspecific, biopsy-proven inflammatory skin changes (interface dermatitis with mucin deposition).

**Fig. 1:**
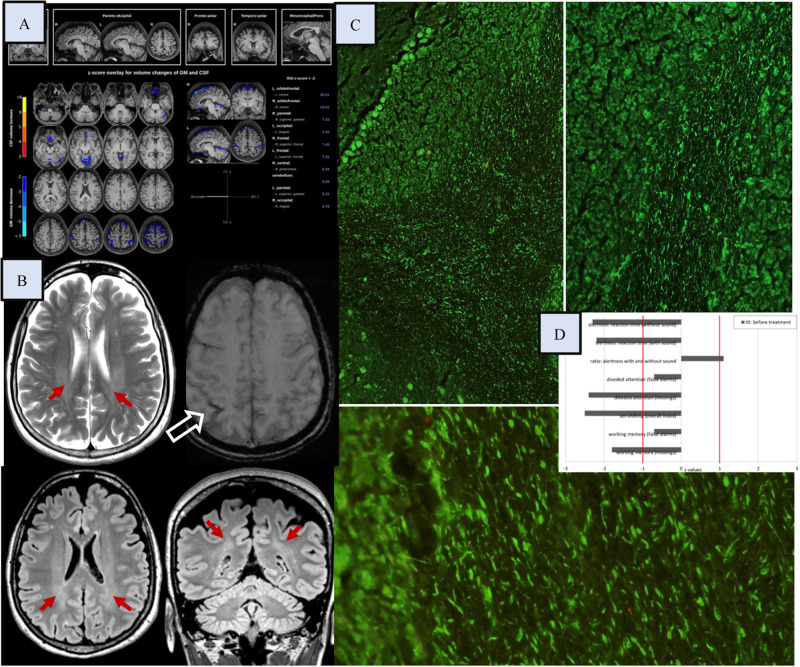
Diagnostic findings: Magnetic resonance imaging (MRI; **A**/**B**) findings, antibody patterns from cerebrospinal fluid (CSF; **C**), and neuropsychological test results (**D**). **A** The automated MRI morphometry (https://www.veobrain.com/?page=veomorph) revealed a relatively global reduction of gray matter (the mean percentage of voxel with z-score < −2 of affected brain regions are shown in blue). **B** The MRI shows some mild periventricular signal increase (red arrows). These findings were suggestive on an incomplete rather than a disturbed myelination. In addition, hemosiderin deposits in the right intraparietal sulcus (open white arrow) as residual of a traumatic subarachnoid hemorrhage were identified. **C** Tissue-based assays on unfixed mouse brain sections using CSF (and serum, not presented here) showed strong immunoglobulin G binding against myelin fibers in the cerebellum [6]. In addition, quantitative profiling of serum/CSF metabolites by liquid chromatography and mass spectrometry was added. Metabolite extraction, quantification, and quality controlling were performed as described in earlier papers [7]. Analysis of neurotransmitters and precursor metabolites in CSF revealed reduced citrate (63 µM; ref. range 176 ± 50 µM), succinate (1.1 µM; ref. range: 29 ± 5 µM), glutamate (4.6 µM; ref. range: 33 ± 7 µM), serine (15 µM; ref. range: 42 ± 15 µM), glutamine (156 µM; ref. range: 440 ± 80 µM), and serotonin (0.018 µM; ref. range 0.82 ± 0.48 µM), as well as slightly elevated GABA (0.213 µM; ref. range 0.1270 ± 0.0052 µM). Dopamine (0.160 nM, ref. range: 0.04–4.5 nM) and 5-hydroxyinolacetic acid (5-HIAA) concentrations (0.127 µM; ref. range: 0.055–0.163 µM) were normal. Analysis of a plasma sample withdrawn on the same day revealed normal concentrations of the above metabolites with the exception of reduced citrate (11 µM; ref. range: 100–150 µM), and elevated concentrations of aromatic amino acids tryptophan (157 µM; ref. range 43–89 µM), and phenylalanine (115 µM; ref. range 28–85 µM; 8,9). **D** The neuropsychological testing for attentional performances showed mainly below-average values (ref. from −1 to +1 z-values).

The diagnostic work-up for possible autoimmune CNS involvement included magnetic resonance imaging (MRI) that revealed some mild periventricular signal increase. In the automated MRI morphometry, a globally reduced brain volume was identified with accentuation in the orbitofrontal, parietal, occipital, frontal, central, and cerebellar regions (https://www.veobrain.com/?page=veomorph). Tissue-based assays on unfixed mouse brain sections using serum and CSF [6] have repeatedly shown strong immunoglobulin (Ig) G binding (+++) against myelin in the cerebellum (Fig. 1). The testing for anti-ganglioside antibodies in serum/CSF showed *borderline positive IgM GM1 antibodies* (IgG GM1 antibodies were negative) only in serum. A wide range of previously characterized anti-myelin and the established well-characterized neuronal antibodies in serum and CSF were all negative [5]. Negative results were also returned for anti-nuclear antibodies (and for tests for extractable nuclear antigens) in the CSF. The routine CSF parameters were normal, including degeneration markers. Research analysis on neurotransmitters in the CSF has revealed reduced serotonin levels [7, 8], reduced glutamate concentrations, and slightly increased GABA [8, 9]. An examination of the gastrointestinal tract revealed bacterial miscolonisation of the small intestine (treated with rifaximin; Supplemental Table 1).

After a multidisciplinary case conference, the patient was given detailed therapeutic options. The patient first preferred classical psychopharmacotherapy, but her BDI showed no change (again 42 points) under venlafaxine and mirtazapine for approximately five weeks. Sertraline had already been tried without effect (each showed good serum levels). Therefore, the patient agreed to try immunotherapeutic treatment, and a low-dose trial with corticosteroids was given starting with 25 mg per day of prednisolone, slowly tapering to 15 mg after six weeks. The BDI showed only slight improvement (36 points) and neuropsychological findings were unchanged, so the steroids were again tapered off. Trimipramine was not well tolerated and did not lead to relevant changes in the BDI (37 points). Pregabalin, which the patient had taken long term, was slowly stopped. With the addition of lithium (and continuation of venlafaxine), the patient showed slight improvement (BDI: 34 points). Under this medication and after intensive psychotherapy, the patient decided she wanted to be discharged (after she was informed about further treatment options).

The biological factors in the case patient with depression included anti-myelin antibodies in the CSF, MRI changes with volume loss and altered myelination, reduced CSF serotonin levels, hyper-inhibitory neurotransmitter changes, bacterial gut miscolonisation, genetic vulnerability, and a systemic autoimmune process.

Initially, autoimmune depression was suspected [3]. Similar anti-myelin antibodies have not previously been detected in patients with depression [5]. The previously characterized IgG antibodies against specific myelin targets—such as MOG, MBP, and MAG—and against anti-ganglioside GM1 all showed negative in the case patient. Interestingly, borderline anti-ganglioside GM1 IgM antibodies were identified in the serum but not in the CSF [10], so a novel myelin-targeting antigen was assumed. The periventricular signal increase in the MRI was suggestive on an incomplete myelination. Impaired myelination is increasingly considered an important factor in the pathophysiology of depression [1]. The specific antibody-binding against cerebellar structures would be compatible with a “cerebellar cognitive affective syndrome” [11]. However, there was no relevant clinical improvement in response to immunotherapy. Therefore, the antibodies could also be irrelevant in the context of an unknown immunological process, representing natural autoantibodies in a susceptible individual or even “reactive” ones due to enormous “brain stress” (after the subarachnoid hemorrhage and alcohol abuse).

At the neurotransmitter level, the CSF finding of reduced serotonin levels were compatible with depressive symptoms [1, 2]. Some studies have found an association between myelin pathologies and serotonin [12], although the exact effects of anti-myelin antibodies on serotonin metabolism remain to be investigated. Therefore, several classical serotonergic antidepressants were started in the patient, which finally yielded a slight improvement. In addition, her marginally elevated GABA concentrations and reduced glutamate levels could reflect a hyper-inhibitory electrophysiological state [13]. Therefore, pregabalin was tapered off in parallel.

A limitation is that the underlying antigen and functionality of the antibodies is unclear, and the examinations did not provide further evidence of neuroinflammation beyond the novel antibodies.

In summary, a multimodal diagnostic work-up may contribute to the understanding of the underlying biological processes in individual patients with depression [1] and thus open the way for precision medicine.

## Supplementary information

Supplemental Table 1

## Data Availability

All necessary data can be found in the paper.
