# Supplementary material for: Depression with anti-myelin antibodies in the cerebrospinal fluid
Source: Mol Psychiatry. 2024 Feb 7;29(6):1909–11. doi: 10.1038/s41380-024-02436-5 (PMC11371637; doi:10.1038/s41380-024-02436-5)
Supplement: Supplementary file 1 — Supplemental Table 1 [file 41380_2024_2436_MOESM1_ESM.pdf]

SUPPLEMENTAL TABLE

**Serum antibodies, immunological markers and serologies**	
Anti-thyroid antibodies (against TPO, TG and TSH-receptor)	Negative
ANAs (*on HEp-2 cells*), ANCAs (*on EthOH-/formalin-fixed neutrophils*), APAs	**Testing for HEp2 resulted in a homogeneous, trace positive finding;** APAs and ANCAs were negative
ENA-differentiation	**Anti-PM-Scl (+++), anti-Mi-2 beta (+), anti-PM-Scl100 (++)**
Complement factors (C3, C4)	Normal
IgG, IgM, and IgA levels	Normal
CRP	<3 mg/l (ref.: < 5 mg/l)
Anti-streptolysin-O	58 IU/ml (ref.: < 300 IU/ml)
Anti-DNaseB	<81 U/ml (ref.: < 200 U/ml)
Serology for Lyme disease or lues	Negative
Serologies (CMV, EBV, HAV, HBV, HCV, HIV, tuberculosis)	**CMV IgG positive (135 U/ml),** CMV IgM negative; **EBV EBNA-1-IgG positive (20.71 S/Co); anti-HBs positive (302 mlU/ml),** anti-HBc negative, Hbs-Ag negative; anti-HCV-IgG negative, HIV negative, TB-antigens 1 and 2 negative
Paraneoplastic IgG antibodies against intracellular antigens	Negative
Well-characterized neuronal IgG cell surface antibodies	Negative
Anti-MOG/AQP4-IgG antibodies	Negative
Tissue based assay on unfixed murine brain tissue (Prof. Prüss; Charité Berlin, Germany)	**IgG binding against myelin in the cerebellum (+++)**
Ganglioside antibody panel (Laboratory Stöcker, Lübeck, Germany)	Ganglioside profile 2 IgG negative, **ganglioside profile 2 IgM identified GM1 antibodies borderline positive**
Myelin IgG antibodies (Laboratory Stöcker, Lübeck, Germany)	"Myelin", MBP, MAG, and GFAP negative
Neurotransmitter measurements (*under medication with sertraline 75 mg and amisulpride 200 mg*)*	Reduced **citrate** (11 µM; ref. range: 100-150 µM), and elevated concentrations of **tryptophan** (157 µM; ref. range 43-89 µM) and **phenylalanine** (115 µM; ref. range 28-85 µM)
**Cerebrospinal fluid**	
White blood cell count	1/µL (ref.: <5/µL)
Protein concentration	227 mg/L (ref.: <450 mg/L)
Albumin quotient	3.6 (ref.: <7.7)
IgG-index	0.49 (ref.: <0.7)
Oligoclonal bands in serum/CSF	Negative/negative
Well-characterized neuronal IgG cell surface antibodies	Negative
Local IgG/IgA/IgM synthesis	0% (ref.: < 10%)
MRZ Reaction	Negative (VZV IgG antibodies were negative in CSF despite the expired multisegmental herpes zoster)
ANAs (*on HEp-2 cells*) and ENA-differentiation	Negative
Tissue based assay on unfixed murine brain tissue (Prof. Prüss, Charité Berlin, Germany)	**IgG binding against myelin in the cerebellum (+++)**
Ganglioside antibody panel (Laboratory Stöcker, Lübeck, Germany)	Ganglioside profile 2 IgG/IgM negative
Myelin antibodies (Laboratory Stöcker, Lübeck, Germany)	"Myelin", MBP, MAG, and GFAP negative
Neurodegeneration markers (Neurochemical Laboratory University of Göttingen, Göttingen, Germany)	Total tau: 97.0 pg/ml (< 450 pg/ml) - normal<br>Phospho-tau: 25.7 pg/ml (< 61 pg/ml) - normal<br>Beta-amyloid ratio: 1.2 (> 0.5) - normal

Neurotransmitter measurements (*under medication with sertraline 75 mg and amisulpride 200 mg*)*	Reduced **citrate** (63 µM; ref. range 176±50 µM), **succinate** (1.1 µM; ref. range: 29±5 µM), **glutamate** (4.6 µM; ref. range: 33±7 µM), **serine** (15 µM; ref. range: 42±15 µM), **glutamine** (156 µM; ref. range: 440±80 µM), and **serotonin** (0.018 µM; ref. range 0.82±0.48 µM), as well as slightly elevated **GABA** (0.213 µM; ref. range 0.1270±0.0052 µM)
**MRI of the neurocranium**	
Visual inspection	**Some mild periventricular signal increase; Hemosiderin deposits in the right intraparietal sulcus as residual of a traumatic subarachnoid hemorrhage;** See also Figure 1
Automated morphometry	See Figure 1
**EEG**	
Visual analyses	No intermittent/ generalized slowing, no epileptic activity
Independent component analysis	Rarely alpha activity at 10.5Hz, no IRDA detections
**FDG-PET**	
Brain	Normal
Whole body	Compared with the previous CT scan, new moderately metabolically increased ***alteration in the partially atelectatic lung middle lobe***
**Ophthalmological tests**	
Optical coherence tomography (OCT)	No OCT abnormalities were evident for both eyes. Both macula and optic disc of both eyes showed normal findings
Electroretinography (ERG)	Flash ERG components (a- and b-wave) for both eyes were normal
**Electrophysiological polyneuropathy screening**	Electromyographically and myosonographically no myopathy, no myositis. Electromyographically, no florid signs of damage, no myopathic changes, ***nonsignificant chronic neurogenic changes***. Myosonographically normal echogenicity of muscle parenchyma, normal vascularization
**Neuropsychological Tests**	
TAP	See Figure 1
MOCA	27/30 points, difficulties in remembering terms; overall result unremarkable
**Psychometric Testing**	
BDI initially	***42***/63 points
**Systemic diagnostic work-up**	
**Heart**	
TTE	No pathological findings
ECG	Sinus rhythm
24h RR	***Borderline elevated blood pressure*** initially
Coronary angiography	Exclusion of a hemodynamically relevant coronary heart disease
**Vessels**	
Blood circulation	***Raynaud's syndrome with a slight reduction in perfusion in the fingers and toes***, polyneuropathy is present; ***megacapillaries*** are detectable in capillary microscopy Hemodynamically relevant peripheral arterial occlusive disease could be excluded
**Lungs/Chest**	
Lung function	***Disturbed diffusion***, no obstruction, no restriction
Bronchoscopy	***Chronic tracheobronchitis***; findings of the BAL gave no evidence of interstitial pulmonary fibrosis
CT chest	***Suspected coronary sclerosis***, in the dorsal right lower lobe polygonally configured compression in the sense of

	a mucus plugging, ***subdiaphragmatic hyperdense mass*** DD of calcified lymph nodes in the context of the underlying autoimmune processes
MRI of the breast	No evidence of malignancy
**Skin**	
Clinical findings	***Dermographism ruber; erythematous brownish plaques on the abdomen and proximal thigh extensor sides***
Biopsy findings	***Interface dermatitis*** with mucin deposition
**Abdomen**	
Sonography of the abdomen	Spleen sonographically not reliably delineated with evidence of a homogeneous isoechoic space dorsal to the kidney, pronounced retention stomach despite fasting
CT abdomen	Except for a smallest (1 mm in diameter) ***renal cyst*** on the left side, no evidence of a mass of the left kidney; spleen in the left upper abdomen, constant for preliminary examination; coprostasis; ***proximal occlusion of the inferior mesenteric artery***
H2/CH4 exhalation test	***Bacterial overgrowth of the small intestine with H2/CH4-forming bacteria***
**Joints**	
Clinical findings	***Rhizarthrosis*** on the right with joint swelling and marked pressure dolence, otherwise no swollen or pressure painful joints
X-rays	***Rhizarthrosis***, stage II according to Eaton and Littler
MRI	***AC joint arthrosis*** on the left with low fluid collection

Supplemental Table 1: All diagnostic findings. ↑ means increased. Conspicuous findings are marked in bold. * *The following neurometabolites were measured: Homocysteine, Cysteine, Cysteamine, Cystathionine, Methionine, Glutathione, Methionine sulfoxide, S-adenosylmethionine, S-adenosylhomocysteine, Creatinine, Argininosuccinic acid, Taurine, Hypotaurine, Homotaurine, Lanthionine, 3-Mercaptopyruvate, Dihydrofolate, 5-Methytetrahydrofolate, Tetrahydrofolate, 5,10-methylene-tetrahydrofolate, Cysteinylglycine, alpha-ketoglutarate, Citrate, Itaconate, Lactate, Malate, Malonate, Methlymalonic acid, Succinate, 2-methylcitrate, Phosphoenolpyruvate, Adenosine, Glucose, Glyceraldehyde-3-phosphate, Glycine, Alanine, Serine, Proline, Valine, Leucine/isoleucine, Aspartic Acid, Lysine, Glutamic Acid, Methionine, Histidine, Arginine, Tryptophan, Tyrosine, Asparagine, Glutamine, Phenylalanine, Threonine, Serotonine, 5-hydroxyinolacetic acid, GABA (gamma-aminobutyric acid), Dopamine, Norepinephrine, Acetylcholine, Choline, and 2-Amino adipic acid. Abbreviations: AC joint, acromioclavicular joint; ANAs, antinuclear antibodies; ANCAs, anti-neutrophil cytoplasmic antibodies; APAs, antiphospholipid antibodies; AQP4, aquaporin-4; DnaseB, deoxyribonuclease B; BDI, Beck Depression Inventory; CMV, cytomegalovirus; CRP, C-reactive protein; CSF, cerebrospinal fluid; CT, computed tomography; EBV, Epstein-Barr virus; ECG, electrocardiography; EEG, electroencephalography; ENAs, extractable nuclear antigens; ERG, electroretinography; FDG-PET, fluorodeoxyglucose-positron emission tomography; HAV, hepatitis A virus; HBV, hepatitis B virus; HCV, hepatitis C virus; HIV, human immunodeficiency virus; IgA/G/M, immunoglobulin A/G/M; IRDA, intermittent rhythmic delta activity; MoCA, Montreal Cognitive Assessment; MOG, myelin oligodendrocyte glycoprotein; MRI, magnetic resonance imaging; MRZ, antibody indices against measles, rubella, and varicella zoster virus; OCT, optical coherence tomography; PCR, polymerase chain reaction; ref., reference; RR, blood pressure; TAP, tests battery for attentional performance; TB, tuberculosis; TG, thyroglobulin; TPO, thyroid peroxidase; TSH, thyroid-stimulating hormone; TTE, transthoracic echocardiography; VZV, varicella zoster virus; WBC, white blood cell.
